# Molecular Imaging of Neuroinflammation in Neurodegenerative Dementias: The Role of In Vivo PET Imaging

**DOI:** 10.3390/ijms18050993

**Published:** 2017-05-05

**Authors:** Chiara Cerami, Leonardo Iaccarino, Daniela Perani

**Affiliations:** 1 Clinical Neuroscience Department, San Raffaele Turro Hospital, Milan 20121-20162, Italy; 2 Division of Neuroscience, San Raffaele Scientific Institute, Milan 20121-20162, Italy; iaccarino.leonardo@hsr.it (L.I.); perani.daniela@hsr.it (D.P.); 3 Faculty of Psychology and Molecular Medicine Doctoral Course, Vita-Salute San Raffaele University, Milan 20121-20162, Italy; 4 Nuclear Medicine Unit, San Raffaele Hospital, Milan 20121-20162, Italy

**Keywords:** neuroinflammation, microglia activation, astrocytosis, molecular imaging, ^11^C-PK11195, TSPO-PET Imaging, Alzheimer’s disease, neurodegenerative disorders

## Abstract

Neurodegeneration elicits neuroinflammatory responses to kill pathogens, clear debris and support tissue repair. Neuroinflammation is a dynamic biological response characterized by the recruitment of innate and adaptive immune system cells in the site of tissue damage. Resident microglia and infiltrating immune cells partake in the restoration of central nervous system homeostasis. Nevertheless, their activation may shift to chronic and aggressive responses, which jeopardize neuron survival and may contribute to the disease process itself. Positron Emission Tomography (PET) molecular imaging represents a unique tool contributing to in vivo investigating of neuroinflammatory processes in patients. In the present review, we first provide an overview on the molecular basis of neuroinflammation in neurodegenerative diseases with emphasis on microglia activation, astrocytosis and the molecular targets for PET imaging. Then, we review the state-of-the-art of in vivo PET imaging for neuroinflammation in dementia conditions associated with different proteinopathies, such as Alzheimer’s disease, frontotemporal lobar degeneration and Parkinsonian spectrum.

## 1. Introduction

Neurodegenerative disorders are due to long-lasting pathological processes associated with the deposition of abnormal toxic proteins aggregates in the brain and the activation of a cascade of aberrant biochemical, metabolic, functional and structural changes [1,2]. Recent literature has focused on the role of neuroinflammatory processes in neurodegeneration [3,4]. In particular, it has been intensely investigated whether neuroinflammation occurs as a primary or secondary event in the course of neurodegenerative disorders, possibly exerting either beneficial/regenerative or detrimental effects [3,4,5,6]. The characteristics of the neuroinflammation response largely depend on the specific features of the trigger insult, including duration and magnitude, which can drive either a neuro-regenerative protective function or a neurotoxic action [7,8].

A significant amount of literature points to a key role for activated microglia and astroglia in neurodegenerative disorders [3,4,5,6,7,8,9]. Presence of activated microglia cells has been demonstrated in the surroundings of amyloid plaques and hyperphosphorylated tau in Alzheimer’s disease (AD) and alpha-synuclein fibrils in Parkinson disease (PD) [4], thus suggesting a biochemical link between the accumulating toxic proteins and microglia activation in neurodegenerative diseases. Comparably, astrocytes play an important role in neurodegenerative disorders with exacerbating effects when they are activated or reactive [9].

Clinical studies have shown that microglia activation, a proxy of neuroinflammation, is present even at the early stages not only in AD and PD, but also in other neurodegenerative conditions such as frontotemporal dementias (FTD) [10,11,12]. Similarly, reactive astrocytosis is an early phenomenon in AD natural history [13,14,15,16]. The possible detrimental role of microglia activation in neurodegenerative disorders and its influence during the different disease phases remain largely unexplored.

Microglia activation and reactive astrocytosis may be assessed in vivo by the use of Positron-Emission Tomography (PET) imaging. The in vivo detection of neuroinflammation could represent a useful tool, not only to identify possible diagnostic signatures, but also to explore the effectiveness of novel treatment targets and monitor the therapeutic efficacy. Additionally, neuroinflammatory responses may considerably influence clinical outcomes as well and the early detection of brain inflammation could help in tailoring individual patient care plans.

This paper reviews the most recent knowledge on in vivo PET imaging of neuroinflammation, focusing on the insights in neurodegenerative diseases.

## 2. Molecular Basis of Neuroinflammation in Neurodegeneration

Microglia cells are tissue-resident mononuclear phagocytes of myeloid lineage, originating from erytromyeloid progenitors formed in the yolk sac, which makes them different from all the other central nervous system (CNS) macrophages formed in the bone marrow [17,18]. Once populated, microglia self-sustain throughout adulthood [19]. When not responding to specific trigger insults, microglia mostly display a ramified morphology and a surveying activity, dynamically scanning the brain parenchyma through continuous retraction and protraction of their highly mobile processes [20]. Microglia in this functional state have been widely referred to as resting or quiescent microglia, notwithstanding the pronounced motility of the surveying activity [20]. In light of this evidence, some authors have referred to them as never-resting cells [21], implying that eventual morphological changes occurring in diseases should be considered as functional switches rather than activation states [20].

The activation of microglia cells is a specific adapting reaction of the brain’s innate immune system, found in both physiological and pathological conditions [22]. Microglia critically contribute to tissue homeostasis, neuronal function and networking, managing to monitor the whole brain parenchyma every few hours [20]. While surveying, whenever microglia cells detect a noxa disturbing tissue homeostasis, they undergo a stepwise transformation towards an effector functional and morphological phenotype [20]. The microglia sensing apparatus, or sensome, involves a unique profile of gene expression, which changes with aging showing upregulation of genes controlling host defense and neuroprotection [23].

In case of direct brain damage, microglia rapidly migrate to the site of brain injury, following directional guidance provided by chemotactic mediators [20]. Microglia functional switching ability and chemotactic reorientation are granted by the expression of multiple surface receptors, binding to neurotransmitters, lipids, neurohormones, cytokines and chemokines [3]. For instance, microglia surface expresses pattern recognition receptors (PRRs) that have the unique ability of sensing either damage-associated or pathogen-associated molecular patterns, i.e., DAMPs and PAMPs [3]. Two major mechanisms account for the activity of these receptors and the subsequent microglia response. First, the detection of specific molecular signaling mediators, i.e., not only microbes or pathogens, but also misfolded and aggregated proteins as those characterizing neurodegenerative diseases. Then, the loss of the so-called “calming inputs” (i.e., the ability to set off the signal) which are usually the result of microglia-neuron crosstalk [20]. A crucial property of microglia activation is indeed represented by its ability of expressing different functional activations according to the stimuli [24]. Nevertheless, the intrinsic microglia reaction is primarily oriented towards pathogen clearance, limitation of brain damage and restoration of tissue homeostasis [3].

Together with activated microglia, the neurodegenerative processes induce the pathological activation of astrocytes and their accumulation particularly around the misfolded protein aggregates (see, for instance, the neuroinflammatory model reported in AD [25]). Hypertrophic reactive astrogliosis is a complex, multistage and pathology-specific reaction, whose effects, comparable to microglia, highly vary in a context-specific manner from adaptive beneficial responses to maladaptive and deleterious processes [26]. A growing body of scientific evidence indicates that the disruption of normal astrocyte functions, together with the astrodegeneration and maladaptive astrogliosis actively contribute to neural dysfunction and neurodegenerative processes [26]. Like microglia, astrocytes are able to release cytokines, interleukins, nitric oxide synthases (NOS), and other potentially cytotoxic molecules after the exposure to toxic proteins such as Aβ protein aggregates in AD [25], thereby exacerbating the neuroinflammatory response.

Depending on the signaling molecules, both microglia activation and reactive astrocytosis can be associated with the production of pro- and anti-inflammatory molecules [3]. For many years, the working hypothesis opposed a classical pro-inflammatory to an alternative anti-inflammatory microglia activation. The former is believed to be associated with possible detrimental neurotoxic effects and exacerbation of damage, caused by excessive release of pro-inflammatory mediators such as reactive oxygen species (ROS), nitric oxide (NO) and other molecules, e.g., interleukin 1β (IL-1β) and tumor necrosis factor α (TNF-α) [3]. The latter is associated with tissue repair and resolution of inflammation, mediated by the production of anti-inflammatory molecules such as interleukin 4 and 10 (IL-4/IL-10) and transforming growth factor 1β (TGF-1β) [3]. These two activation states were named *M1* and *M2*, adopting a nomenclature associated with the functional properties of effector T helper cells (T_H_), which express a specific reaction when stimulated with pro-inflammatory (interferon γ, IFN-γ), i.e., T_H_1 cells, or anti-inflammatory mediators, i.e., T_H_2 cells [27]. Recent stances and evidence, however, have challenged this working hypothesis, which is now considered over simplistic [9,28]. This is especially true for the multiple functional phenotypes that microglia can adopt, which can dynamically switch in accordance with changes in the environment, thus delineating a very complex and dynamic system [9,24,28].

In neurodegenerative proteinopathies such as AD, the leading hypothesis postulates that the initial activation of microglia cells and astrocytes is an attempt to clear the protein aggregates (neuroprotective effect). However, due to factors specific to the misfolded proteins, glial cells are unable to clear efficiently the environment. The prolonged exposition to the trigger insult and its increasing burden lead to a chronic activation of microglia and astrocytes (neurotoxic effect), which is thought to ultimately contribute to neuronal dysfunction [3,4,5,6,9]. The neuroinflammation may thus be an additional pathological component, which, once initiated, actively contributes to neurodegeneration and disease progression [25].

Such local immune activation and toxic protein accumulation can also be modulated by specific modifiers, such as *TREM2* gene polymorphisms, and risk factors for neurodegenerative disorders, including AD [29,30,31], PD and FTD [32].

## 3. PET Molecular Imaging of Neuroinflammation

During activation, functional and morphological changes of microglia are coupled to the over-expression or de novo expression of several receptors [9]. This property paved the way for the flourishing of PET-based molecular imaging techniques targeting neuroinflammation [33,34]. The majority of PET radioligands developed for the use in humans currently targets the over-expression of the 18 kDa Translocator Protein (i.e., TSPO, formerly known as Peripheral Benzodiazepine Receptor-PBR). Other microglia activation ligands measure cannabinoid and purinergic receptors, whereas astrocytes activation can be measured by targeting the monoamine oxidase B (MAO-B) enzyme [33,34].

The TSPO is an outer mitochondrial membrane protein that is well known to be over-expressed in microglia activation, thus being a sensitive hallmark of neuroinflammation [35,36,37]. Under normal conditions, levels of TSPO are low in the central nervous system. In response to injury, TSPO expression is markedly increased, mostly in reactive microglia and, to a lower extent, in astrocytes [38]. On the contrary, the MAO-B enzyme, which is localized on the outer mitochondrial membrane, occurs predominantly in astrocytes [39]. The distribution of TSPO and MAO-B, however, is highly variable depending on disease, disease phase and proximity to the lesion [39,40].

A number of ligands have been developed for the in vivo visualization and measurement of TSPO over-expression. ^11^C-PK11195 is by far the most validated and adopted in human studies [41]. It has been used to explore patterns of neuroinflammation both in healthy subjects and in neurological diseases, including neurodegenerative conditions [10,42,43,44,45,46]. ^11^C-PK11195 presents, however, some limits, such as highly lipophilic nature, low bioavailability, high non-specific binding and limited capacity to detect small changes in TSPO expression, which led to a recent effort towards the development of second-generation TSPO ligands [41]. These new generation tracers include both carbon-11 and fluorine-18 radioligands, such as ^11^C-DPA713 [47], ^11^C-DAA1106 [48], ^11^C-PBR28 [49,50,51], ^11^C-vinpocetine [52], ^18^F-DPA714 [53], ^18^F-FEPPA [54], ^18^F-FEMPA [55] and ^18^F-FEDAA1106 [56,57,58,59,60], which have all been tested in a few human studies. Notably, TSPO genotype may considerably influence the second-generation radiotracer binding affinity [59], making genetic testing mandatory. Different from second-generation tracers, ^11^C-PK11195 binding does not seem to be influenced by the TSPO polymorphism [61].

At present, ^11^C-PK11195 remains thus the used and characterized TSPO ligand [41], with possible promising applications for the monitoring of anti-inflammatory therapies [60].

TSPO PET techniques, irrespectively of the radioligand, share several caveats, which are intrinsically related to the target protein [62]. These include: (i) the considerable amount of TSPO in the endothelium; (ii) the variability of plasma free fractions across human studies; and (iii) the presence of TSPO genetic polymorphism [61] that can alter radioligand binding [62].

The semi-quantification of TSPO-based PET signal can be particularly challenging due to the biological distribution of TSPO [62]. More specifically, TSPO distributes rather homogeneously across the whole-brain, thus the resulting images hinder the selection of an anatomically defined reference region [63]. The characteristics of this molecular target have fostered the development of advanced voxel clustering approaches [63,64]. These approaches are based on the modelling of single–voxel Time Activity Curves (TAC), compared with a pre-defined set of kinetic classes related to different tissue compartments, such as white and grey matter [63,64]. These algorithms thus deliver subject-specific pseudo-reference regions, which are groups of voxels sharing TACs typical of grey matter without specific binding [63,64]. Given the high TSPO binding in endothelium and at the blood–brain barrier (BBB) level, the integrity of which might be deranged in neurodegeneration, vasculature TSPO binding also needs to be accounted for to obtain an optimal specific binding estimation [62,65,66].

In addition, several neuroimaging studies have employed a specific neuroinflammation radioligand, i.e., ^11^C-deuterium-l-deprenyl or ^11^C-DED, which specifically targets astrocytes. This molecule is an irreversible MAO-B inhibitor with high affinity and specificity for this enzyme, predominantly expressed on the outer mitochondrial membrane of astrocytes [39]. Thus far, PET studies using ^11^C-DED have been performed in some neurological diseases including amyotrophic lateral sclerosis, Creutzfeldt-Jakob disease [67,68,69] and AD [13,14,15,16].

Additional new targets for the in vivo detection of neuroinflammation in humans are currently under evaluation [42]. These include purinergic receptors (e.g., P2X7), cannabinoid receptors (e.g., type 2 receptor or CB_2_R), metalloproteinases, cyclooxygenase enzymes (i.e., COX-1 and COX-2) which are all expressed by monocytes and macrophages immune cells, and arachidonic acid (AA) whose metabolism is upregulated by inflammatory cytokines and nitric oxide released from microglia and astrocytes.

Different from the expected findings, a recent ^11^C-NE40 PET study has shown no in vivo CB_2_R upregulation in AD patients and no regional co-localization to Aβ deposits [70]. The authors thus argued for a possible lower affinity of the radioligand (^11^C-NE40) to CB_2_R [70]. A pilot PET study with ^11^C-radiolabeled AA showed in few AD patients a widely elevated tracer uptake in different neocortical regions [71].

## 4. In Vivo Pet Evidence in Neurodegenerative Dementias

### 4.1. Alzheimer’s Dementia and Prodromal Alzheimer’s Disease

The majority of PET imaging in neuroinflammation research focused on the study of microglia activation in AD patients [10,42]. Based on solid preclinical and clinical data showing that immune system-mediated actions actively contribute to and drive AD pathogenesis [72], there was an increasing interest on the role of neuroinflammation within the pathological mechanisms occurring during the course of the disease [25]. As for Aβ peptides deposition and neurofibrillary tangles formation, the key AD pathological hallmarks, neuroinflammation is now considered as a third component, which actively contributes to disease progression and chronic degeneration [25].

**Figure 1:**
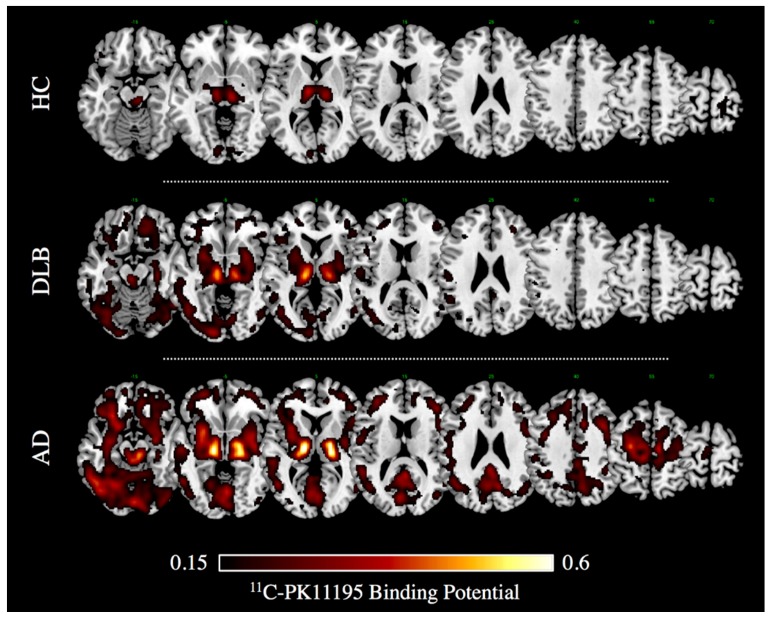
^11^C-PK11195 Binding Potential images in a case of dementia with Lewy bodies (DLB, 75 years old, Male) and Alzheimer’s dementia (i.e., AD, 74 years old, Male), and in an age-matched healthy volunteer (i.e., HC). Patients gave a full informed consent to PET studies. Multiple axial views are overlaid on a standard anatomical template with MRIcron software. A minimum threshold was adopted for the sake of better visualization. (Image courtesy Daniela Perani, Nuclear Medicine Unit, San Raffaele Hospital, Milan, Italy).

About two thirds of reports in literature investigated microglia activation with TSPO PET imaging [10,42]. First, in vivo PET evidence of microglia activation in AD dates back to two decades ago [73]. Notwithstanding some negative imaging, findings questioned the role of microgliosis as the inciting pathophysiologic factor in AD [52,58,64,74,75], and the majority of PET studies reveals significant activation of microglia in mild-moderate AD patients [10]. In vivo measurements of microglia activation with ^11^C-PK11195 PET imaging indeed showed increased regional tracer binding in the entorhinal, temporo-parietal, and cingulate cortex in AD and also in mild cognitive impairment (MCI) subjects [76], indicating that AD pathology could be associated with an early inflammatory response. Several studies confirmed an increased ^11^C-PK11195 uptake in most AD patients and MCI subjects compared with controls in different neocortical regions, with the largest effect in the temporo-parietal cortex [66,77,78,79,80,81,82,83,84] (see Figure 1). The results in MCI subjects, however, vary across studies, mostly depending on the method used for the quantification of the ^11^C-PK11195 uptake [76,83,84].

The topic of a possible association between neuroinflammation and amyloid burden is currently being debated. Some studies reported an inflammatory pattern mirroring the pattern of amyloid plaque deposition in both AD [78,79,84] and prodromal AD (i.e., MCI) [82]. Other authors found opposite results [75]. Crucially, it has been reported that neuroinflammation may occur independently of amyloid burden, especially in the later AD stages [83].

Notably, cognitive status significantly correlated with levels of cortical microglia activation, but not with amyloid load, suggesting the active role of cortical neuroinflammation in driving neuronal dysfunction [78,79,84]. Accordingly, combined PET studies provided evidence for a significant inverse correlation between microglia activation and glucose metabolism in AD, MCI and PD dementia (PDD) patients [79] as well as with hippocampal volume in AD and PDD [80]. In particular, in parallel with a progressive reduction of the medial temporal lobe volume, the majority of AD patients shows an increase of microglia activation in the same region over time (i.e., at a 16-month PET follow-up) [81].

Recent literature focused on second-generation ligands. In vivo human studies on ^11^C-PBR28 and ^18^F-DPA714 tracers showed promising results on AD. Kreisl et al. [51] reported higher ^11^C-PBR28 tracer uptake in temporo-parietal regions particularly in the later stages of AD rather than in early AD, with an increase from 3.9% to 6.3% per annum in patients, while they showed no uptake in MCI [50,51], thus suggesting a role of ^11^C-PBR28 PET imaging as biomarker of AD progression [51]. A recent large ^18^F-DPA714 PET study shows remarkable results in prodromal AD [53]. Here, the use of TSPO genotyping and semi-quantitative analysis of PET data allowed for obtaining solid results even in early AD phase indicating presence of neuroinflammation [53]. Moreover, both ^18^F-DPA714 and ^11^C-PBR28 uptake correlated with cognitive status and grey matter volume [51,53]. Today, the reported findings from PET with other second-generation tracers (i.e., ^18^F-FEPPA, ^18^F-FEMPA, ^18^F-FEDAA1106 and ^11^C-DAA1106) are sparse and contradictory [48,54,55,56,57,58,85]. None of the second-generation tracers at present has sufficient and solid evidence to support their use in human studies.

Comparative TSPO PET studies (i.e., first- vs. second-generation tracers) are still lacking. The only one comparative PET study (i.e., ^11^C-DPA713 vs. ^11^C-PK11195) showed a greater sensitivity to TSPO activation for the second-generation tracer in both aging and AD patients [47]. The authors reported also significant correlation between cognitive score and ^11^C-DPA713 binding potential in several brain regions, whereas ^11^C-PK11195 failed to correlate with neuropsychological scores in this series [47]. These findings should be considered as preliminary and need to be confirmed in larger studies.

Reactive astrocytosis has been investigated in vivo only recently through the use of ^11^C-DED tracer [13,14,15,16]. Significant tracer retention in the frontal, parietal and temporal cortices was observed compared to healthy controls in both moderate-severe AD patients [13] and ^11^C-Pittsburgh Compound B (^11^C-PIB) positive MCI [14]. This latter finding suggests reactive astrocytosis as early events in AD. Moreover, positive correlation between high ^11^C-DED uptake in the parahippocampus and grey-matter loss in amyloid-positive MCI subjects suggest also an early influence of astrocytosis on cellular tissue loss [15]. Additionally, Santillo et al. reported a regional correlation between ^11^C-DED and amyloid burden [13]. This was not confirmed by following multitracer PET studies [14,15]. Notably, a large longitudinal multitracer PET study (i.e., ^11^C-DED, ^11^C-PIB and ^18^F- Fludeoxyglucose (^18^F-FDG)) on genetic and sporadic AD patients showed divergent patterns of amyloid deposition and astrocytosis [16]. While ^11^C-DED uptake was significantly elevated only in the early presymptomatic stages in genetic AD cases, amyloid-β plaque deposition increased with disease progression [16]. There was a significant longitudinal reduction in astrocytosis over time both in genetic and sporadic AD cases [16]. Thus, the authors indicate the role of astrocyte activation, especially in the very early stages of AD pathology.

Notwithstanding that PET research has largely investigated microglia activation in AD dementia, only a few studies explored possible differential patterns of TSPO binding according to the AD variants. Kreisl et al. [49] showed greater ^11^C-PBR28 binding in posterior cortical atrophy-PCA in occipital, posterior parietal and temporal regions, notably with a close mirroring of the hypometabolic pattern revealed by ^18^F-FDG PET imaging [86]. In contrast, typical AD (i.e., amnestic AD) patients showed greater tracer binding in inferior and medial temporal cortex [50]. In addition, these authors reported greater ^11^C-PBR28 binding in early-onset AD (<65 years) than late-onset patients [51], particularly in parietal cortex and striatum in which tracer binding correlated with age of onset. These results suggest that microglia activation is a mark close to neurodegeneration across different subtypes of AD.

### 4.2. Frontotemporal Lobar Degeneration and Parkinsonisms

In the last years, some pilot PET studies investigated the microglia activation in vivo with PET imaging in frontotemporal lobar degeneration (FTLD) patients [87,88,89] as well as in presymptomatic FTLD mutated carriers [11,12]. Cagnin et al. [87] reported a distribution pattern of microglia activation mirroring the pattern of neuronal metabolic dysfunction and clinical impairment in five patients fulfilling Neary criteria for FTD [90]. The group analysis revealed increased ^11^C-PK11195 uptake in the dorsolateral prefrontal cortex, the hippocampal structures and putamen bilaterally [87]. Notably, there was a reported asymmetric left-sided pattern resulting from group analysis [87], likely due to the clinical presentation of some included patients (i.e., four patients presented with aphasic non-fluent variant and one with a behavioral variant).

There is also in literature two preliminary studies in five corticobasal degeneration (CBD) patients and four progressive supranuclear palsy (PSP) supporting increases of microglia activation associated with neuronal dysfunction and corresponding with the reported topographical distribution of neuropathology changes in these two degenerative conditions [89]. Namely, high ^11^C-PK11195 binding was found in the caudate, putamen, substantia nigra, and pons, pre- and postcentral gyri and in the frontal lobe in CBD patients [88] and in the basal ganglia, midbrain, frontal cortex and in the cerebellum in PSP patients [89].

Neuroinflammation has been also investigated using ^11^C-PK11195 and PET in another atypical Parkinsonism, i.e., dementia with Lewy bodies (DLB) [91]. In this study, increased tracer uptake was found in early DLB compared to PD patients, even at a single subject level, with commonalities and differences in the topographical distribution of microglia activation [91]. Microglia activation in the nigro-striatal pathway (i.e., substantia nigra and putamen) was reported in both PD and DLB cases [91]. Notably, a severe cortical neuroinflammation was found in DLB patients in several associative cerebral cortices, including the occipital [91] (see Figure 1), while only in a few PD cases there was an increased TSPO tracer uptake limited to the anterior cingulate and prefrontal cortex [91]. This evidence is in line with other comparable findings showing different patterns of TSPO tracer uptake selectively involving basal ganglia, midbrain, and frontal regions in PD [92,93,94].

Finally, only two studies investigated genetic neurodegenerative dementias. A study investigated microglia activation, striatal dopaminergic function and acetylcholinesterase with PET imaging as well as morphologic brain changes with MRI in three presymptomatic carriers of mutation on *MAPT* genes, belonging to kindred affected by frontotemporal dementia with Parkinsonism linked to chromosome 17 (FTDP-17) [11]. In this study, ^11^C-DAA1106 PET revealed significantly higher uptake in the frontal, occipital and posterior cingulate cortices in carriers compared to healthy volunteers [11]. This supports the presence of neuroinflammation in genetic tauopathies yet in a very early disease phase. More recently, Lant et al. [12] explored whether TSPO PET imaging could be used as diagnostic biomarker able to differentiate FTLD from AD and according to the different histological subtype [12]. These authors reported indeed significantly higher levels of microglia activation in fronto-temporal cortex in FTLD patients compared to controls and in frontal subcortical white matter in FTLD compared to AD subjects [12]. In addition, *MAPT* mutated FTLD cases showed significantly higher levels of tracer binding in the temporal subcortical white matter than in other genetic (i.e., *GRN*, *C9ORF72*) or non-genetic FTLD cases [12].

Positive PET findings in AD and FTLD cases support the hypothesis that glial activation is a generic pathological response in neurodegenerative dementias linked to the process of neuronal degeneration rather than to the deposition of a specific toxic protein. This process, however, is likely to occur early in the disease course, especially in the genetic tauopathies.

## 5. Conclusions

Emerging evidence suggests that neuroinflammation is a highly dynamic event with a potential pathogenetic role in neurodegenerative disorders [1]. Further large studies are required in order to establish the pathological role of neuroinflammation in neurodegenerative disorders. A better characterization of the glial responses in vivo at the individual level will also allow for improving the understanding of the pathophysiology of the neurodegenerative disorders and possibly identifying and monitoring new therapeutic targets.

In vivo PET molecular imaging methods are continuously improving, providing validated and quantitative molecular data in neurology. PET molecular imaging of neuroinflammation, although still having some methodological caveats, may represent a valid method for in vivo quantification of resident glial activation, useful for investigating neuropathology but also for testing novel therapies (see, for instance, [95,96,97,98,99]). Crucially, PET imaging gives the opportunity to explore neuroinflammatory changes in individual patients in terms of both timing and spatial distribution, in order to evaluate the functional consequences and eventually guide the development and assessment of new therapies or even monitor the effectiveness of anti-inflammatory interventions [60]. In this framework, TSPO-PET imaging is at present the most reliable tool to detect activated microglia in dementias and neurodegenerative disorders in general. Some studies focused on testing anti-inflammatory drugs in neurological disorders adopted TSPO-PET imaging to assess pre- and post-treatment changes in brain inflammatory patterns [60,98,99]. Although based on relatively small samples, these preliminary reports highlighted the great potential of PET imaging techniques in clinical trials.

Despite its great potential, PET imaging of brain inflammation is still in its early days. Literature evidence to date does not clearly demonstrate whether the neuroinflammation differs between dementia phenotypes (e.g., typical AD vs. atypical AD) or whether the neuroinflammatory patterns are reproducible between disease stages (e.g., prodromal AD vs. mild/advanced AD). Therefore, while in the last two decades compelling evidence for TSPO over-expression in disease-specific regions in multiple neurodegenerative conditions has been provided [25,41], heterogeneity in both results and methodology limits the use of TSPO-PET imaging in clinical settings. Additional evidence is needed to consistently assess the diagnostic and prognostic value of TSPO-PET imaging. The translation of these techniques in routine clinical practice will certainly require the improvement of methodological issues surrounding TSPO imaging [100]. In this direction, the recent development of second- and third-generation TSPO fluorine-18 radioligands is promising and is likely to favor multi-site evaluations, overcoming the limits imposed by the short half-life of carbon-11 compounds. Still, different from first- and second-generation carbon-11 ligands, only a few studies employing novel fluorine-18 radioligands are currently available in literature, urging for a thorough evaluation of these techniques in clinical practice [100]. Another key issue regards the standardization of TSPO-PET methodology, since its quantification is known to be particularly challenging [62], also considering the influence of TSPO genotype [59] as well as TSPO tissue and cellular distribution [62]. Guidelines for a data analysis approach are definitely needed and will improve PET neuroinflammation imaging, e.g., a metabolite-corrected plasma input model (classical two-tissue compartment model), simple reference tissue modelling with clustering approaches to select reference regions, or simple standardized uptake value ratios (SUVr) with anatomical reference tissue regions to measure the cortical tracer binding [62].
